# The Complete Genome Sequence of *Escherichia coli* EC958: A High Quality Reference Sequence for the Globally Disseminated Multidrug Resistant *E. coli* O25b:H4-ST131 Clone

**DOI:** 10.1371/journal.pone.0104400

**Published:** 2014-08-15

**Authors:** Brian M. Forde, Nouri L. Ben Zakour, Mitchell Stanton-Cook, Minh-Duy Phan, Makrina Totsika, Kate M. Peters, Kok Gan Chan, Mark A. Schembri, Mathew Upton, Scott A. Beatson

**Affiliations:** 1 Australian Infectious Diseases Research Centre, School of Chemistry & Molecular Biosciences, The University of Queensland, Queensland, Australia; 2 Division of Genetics and Molecular Biology, Institute of Biological Sciences, Faculty of Science, University of Malaya, Kuala Lumpur, Malaysia; 3 Plymouth University Peninsula Schools of Medicine and Dentistry, Plymouth, United Kingdom; University of Münster, Germany

## Abstract

*Escherichia coli* ST131 is now recognised as a leading contributor to urinary tract and bloodstream infections in both community and clinical settings. Here we present the complete, annotated genome of *E. coli* EC958, which was isolated from the urine of a patient presenting with a urinary tract infection in the Northwest region of England and represents the most well characterised ST131 strain. Sequencing was carried out using the Pacific Biosciences platform, which provided sufficient depth and read-length to produce a complete genome without the need for other technologies. The discovery of spurious contigs within the assembly that correspond to site-specific inversions in the tail fibre regions of prophages demonstrates the potential for this technology to reveal dynamic evolutionary mechanisms. *E. coli* EC958 belongs to the major subgroup of ST131 strains that produce the CTX-M-15 extended spectrum β-lactamase, are fluoroquinolone resistant and encode the *fimH30* type 1 fimbrial adhesin. This subgroup includes the Indian strain NA114 and the North American strain JJ1886. A comparison of the genomes of EC958, JJ1886 and NA114 revealed that differences in the arrangement of genomic islands, prophages and other repetitive elements in the NA114 genome are not biologically relevant and are due to misassembly. The availability of a high quality uropathogenic *E. coli* ST131 genome provides a reference for understanding this multidrug resistant pathogen and will facilitate novel functional, comparative and clinical studies of the *E. coli* ST131 clonal lineage.

## Introduction

Many multidrug resistant (MDR) *Escherichia coli* strains belong to specific clones that are frequently isolated from urinary tract and bloodstream infections. These clones may originate in a specific locale, country or may be distributed globally without a clear place of origin. A major contributor to this phenomenon is *E. coli* ST131, a group of *E. coli* strains of multi-locus sequence type 131 (ST131) that have emerged rapidly and disseminated globally in hospitals and the community, causing MDR infections typically associated with frequent recurrences and limited treatment options [1]–[4]. *E. coli* ST131 strains are commonly identified among *E. coli* producing the CTX-M-15 type extended-spectrum β-lactamase (ESBL), currently the most widespread CTX-M ESBL enzyme worldwide [1], [4], [5]. The largest sub-clonal lineage of *E. coli* ST131 is resistant to fluoroquinolones and belongs to the *fimH*-based *H*30 group [6].

*E. coli* EC958 represents one of the most well characterised *E. coli* ST131 strains in the literature. *E. coli* EC958 is a phylogenetic group B2, CTX-M-15 positive, fluoroquinolone resistant, *H*30 *E. coli* ST131 strain isolated from the urine of an 8-year old girl presenting in the community in March 2005 in the United Kingdom (UK) [7]. The strain belongs to the pulse field gel electrophoresis defined UK epidemic strain A and has a O25b:H4 serotype [8]. *E. coli* EC958 contains multiple genes associated with the virulence of extra-intestinal *E. coli*, including those encoding adhesins, autotransporter proteins and siderophore receptors. *E. coli* EC958 expresses type 1 fimbriae and this is required for adherence to and invasion of human bladder cells, as well as colonization of the mouse bladder [7]. In mice, *E. coli* EC958 causes acute and chronic urinary tract infection (UTI) [9], as well as impairment of ureter contractility [10]. *E. coli* EC958 bladder infection follows a well-defined pathogenic pathway that involves the formation of intracellular bacterial communities (IBCs) in superficial epithelial cells and the subsequent release of rod-shaped and filamentous bacteria into the bladder lumen [9]. *E. coli* EC958 also causes impairment of uterine contractility [10], and is resistant to the bactericidal action of human serum [11]. The complement of genes that define the serum resistome of *E. coli* EC958 have been comprehensively defined [11].

Second generation sequencing (SGS) technologies have revolutionised genome research through the provision of a rapid, cost-effective method for generating sequence data. However, obtaining complete bacterial genomes using these technologies has been challenging. Short read lengths are a characteristic feature of SGS technologies and highly repetitive stretches of DNA, often present in multiple copies, are difficult to correctly resolve using these platforms. Typically, these assemblies are highly fragmented, prone to misassembly and require costly and time consuming finishing procedures [12]–[14]. Consequently, most genomes are not completely resolved; they are submitted as draft genomes, often containing hundreds of contigs that are generally unannotated or poorly annotated [15]. As a result, many of these genomes are of limited use for comparative, functional, clinical and epidemiological studies [16]. In contrast to other methods, the Pacific Biosciences (PacBio) single molecule real time (SMRT) sequencing platform [17] can produce read lengths of up to 30,000 bp that are capable of spanning large repeat regions (such as rRNA operons), thereby facilitating the generation of complete genome assemblies without the need for additional sequencing.

In order to enhance our knowledge of *E. coli* ST131 and its capacity to cause disease, a greater understanding of this clone is required at the genomic level. Four complete or draft *E. coli* ST131 genome sequences are currently available, namely EC958 (draft) [7], SE15 [18], NA114 [19] and most recently JJ1886 [20]. EC958, NA114 and JJ1886 are all phylogroup B2, CTX-M-15 positive, fluoroquinolone resistant, *H*30 strains which have recently been shown in two independent phylogenomic studies to belong to single clade (ST131 clade C) distinct from SE15 (ST131 clade A) [6], [21]. A pair-wise comparison between SE15 and NA114 demonstrated that SE15 contains a number of differences in genome content despite being closely related at the core genome level [22]. Furthermore, we have shown that many of the genomic islands and prophage regions previously identified in the draft EC958 genome [7] are well conserved in most other fluoroquinolone resistant, clade C/*fimH*30 strains [21]. Here we used PacBio SMRT sequencing to determine the complete genome sequence of *E. coli* EC958. The *E. coli* EC958 genome represents as an accurate reference for future functional, comparative, phylogenetic and clinical studies of *E. coli* ST131.

## Methods

### Genome sequencing and assembly

Genomic DNA for *E. coli* EC958 was prepared using the Qiagen DNeasy Blood and Tissue kit, as per manufacturer's instructions. The genome of *E. coli* EC958 was sequenced by generating a total of 601,224 pre-filtered reads with an average length of 1,600 bp, from six SMRT cells on a PacBio RS I sequencing instrument, using an 8–12 kilobase (kb) insert library, generating approximately 200-fold coverage (GATC Biotech AG, Germany).

*De novo* genome assemblies were produced using PacBio's SMRT Portal (v2.0.0) and the hierarchical genome assembly process (HGAP) [23], with default settings and a seed read cut-off length of 5,000 bp to ensure accurate assembly across *E. coli* rRNA operons. Assemblies were performed multiple times using different combinations of between one and six SMRT cells of read data. The best assembly results were obtained with six SMRT cells which yielded approximately 547 Mb of sequence from 190,145 post-filtered reads (Table 1). The average read length was found to be 2,875 bp with an average single pass accuracy of 86.5%. During the preassembly stage 190,145 long reads were converted into 23,772 high quality, preassembled reads with an average length of 4,573 bp. Assembly of these reads returned seven contigs, three were greater than 500 kb. Furthermore, the largest contig (∼3.8 Mb) was estimated to contain 74.5% of the chromosome of EC958. For all other assemblies total contig numbers exceeded 10 (Table 1). However, for assemblies using two or three SMRT cells, assembly metrics could be improved >2-fold by reducing the seed read length (Table 1).

**Table 1:** PacBio assembly statistics.

		Raw read data			Pre-assembly		Final assembly		
SMRT cells	Seed length^1^	Total bases^2^	Total reads	Average length^3^	Total bases^2^	Total reads	Assembly size^3^	Total contigs	N50
1	5	89	33736	2649	7	2381	1372346	162	8748
2	5	177	63802	2913	29	6244	5163106	154	56927
2	1.5	177	63802	2777	91	37720	5262395	44	225550
3	5	286	97231	2945	47	10407	5298899	40	216859
3	2.7	268	96187	2793	105	31531	5317490	20	594137
4	5	383	130044	2946	65	13934	5311243	18	1061190
4	3.5	357	125866	2844	108	27592	5314416	17	769937
5	5	472	159723	2958	81	17175	5320054	14	1100290
5	4.1	449	157332	2859	108	25345	5339571	16	710956
6	5	546	190145	2875	108	23772	5298989	7	3866706

1 Kilobase-pairs;

2 Megabase-pairs;

3 Base-pairs.

To determine their correct order and orientation, contigs from our six SMRT cell assembly were aligned to the complete genome of *E. coli* SE15 using Mauve v. 2.3.1 [24]. Contig ordering was confirmed by PCR. Overlapping but un-joined contigs, a characterised artefact of the HGAP assembly process [23], were manually trimmed based on sequence similarity and joined. All joins were manually inspected using ACT [25] and Contiguity (http://mjsull.github.io/Contiguity/).

A single contig representing the EC958 large plasmid pEC958 was identified and isolated by BLASTn comparison against the previous draft assembly of EC958 (NZ_CAFL00000000.1) [7]. Overlapping sequences on the 5′ and 3′ ends of the plasmid contig were then manually trimmed based on sequence similarity. Although the EC958 small plasmid (pEC958B) was too small to be assembled as part of the main assembly, 25 unassembled PacBio reads, with an average length of 2,031 bp, were found to align to the small 4,080 bp plasmid contig that had previously been assembled from 454 GS-FLX reads (emb|CAFL01000138).

To determine if reads containing unremoved adapter sequence have had an impact on the assembly of EC958 we first screened the filtered subreads for adapter sequence using BBMap version 31.40 (http://sourceforge.net/projects/bbmap/). A high level of adapter contamination would likely pose some risk of misassembly. Additionally, to eliminate the possibility that aberrant reads have resulted in the inclusion of assembly artefacts in the EC958 genome assembly, contig-ends were screened for hairpin artefacts using MUMmer version 3.23 [26].

### Genome annotation and comparison

Initial annotation of the genome of EC958 was done by annotation transfer from the draft genome of EC958 (NZ_CAFL00000000.1) using the rapid annotation transfer tool (RATT) [27]. In addition, the genome of EC958 was subject to additional automatic annotation using Prokka (Prokka: Prokaryotic Genome Annotation System - http://vicbioinformatics.com/). All predicted protein coding sequences were searched (BLASTp) against the reannotated genome of *E. coli* UTI89 [28], [29] with the aim of correcting CDS start sites and assigning correct gene names and an appropriate functional annotation. Whole genome nucleotide alignments for *E. coli* EC958, SE15 and NA114 were generated using BLASTn and visualised using Easyfig version 2.1 [30], Artemis Comparison Tool [25] and BRIG [31]. To compare the original 454 draft genome and the complete PacBio genome, 454 sequencing reads used for the draft assembly of *E. coli* EC958 [7] were mapped to the complete *E. coli* EC958 genome using SHRiMP v 2.0 [32]. SNP calling and insertion/deletion (indel) prediction were performed using the Nesoni package with default parameters (http://www.vicbioinformatics.com/software.nesoni.shtml). Additional platform-specific SNPs and indels were identified by comparison of the 454 draft genome contigs and the PacBio complete genome using MUMmer 3.23 [26]. The complete annotated chromosome of EC958, large plasmid (pEC958A) and small plasmid (pEC958B) are available at the European Nucleotide Archive (ENA; http://www.ebi.ac.uk/ena) under the accession numbers HG941718, HG941719 and HG941720 respectively.

### Phylogenetic analysis

To determine the phylogenetic relatedness of the four complete ST131 genomes, a single-nucleotide polymorphism (SNP) based phylogenetic tree was constructed. The pan-genome SNPs in EC958, 3 complete ST131 genomes (*E. coli* SE15, NA114 and JJ886), an additional 16 representative complete *E. coli* genomes: *E. coli* ED1A, CFT073, UTI89, 536, S88, APEC-01, IAI39, UMN026, HS, W3110, MG1655, BW2952, IAI1, SE11, Sakai, EDL933 [20], [28], [33]–[42] and the out-group species *E. fergusonii* ATCC35469 were identified using kSNP2 2.1.1 [43] (using default setting and a k-mer size of 21). In total, 261,214 SNPs were found to be common to all 21 *E. coli* genomes, including EC958. SNPs in each genome were concatenated into single contiguous sequences and aligned. The resulting SNP-based alignment was used for phylogenetic analysis. A maximum likelihood (ML) phylogenetic tree was constructed with PhyML 3.0 [44], using the GTR nucleotide substitution model and 1000 bootstrap replicates. The phylogenetic tree was plotted using FigTree 1.4.0 (http://tree.bio.ed.ac.uk/software/figtree/).

### Genome assembly of EC958 using simulated Illumina paired-end reads

In an attempt to replicate the assembly protocol of *E. coli* NA114, simulated Illumina sequencing and assembly of *E. coli* EC958 was performed as described for *E. coli* NA114 in Avasthi et al [19]. The chromosome of EC958 was used as a reference to generate 500-fold coverage of simulated 54 bp, error free, Illumina paired-end reads with an average insert size of 300 bp. These simulated Illumina paired-end reads were then assembled using Velvet 1.2.7 [45]. Assembled contigs were ordered and orientated by aligning them to the genome of *E. coli* SE15 using Mauve and concatenated to produce a ∼5 Mb pseudo-molecule.

## Results

### The complete PacBio genome assembly of *E. coli* EC958 reveals dynamic phage rearrangements

To determine the complete genome sequence of *E. coli* EC958 we carried out sequencing of genomic DNA using the PacBio RS I platform. An initial assembly of seven contigs representing the *E. coli* EC958 genome was produced by HGAP [21] using 190,145 post-filtered reads from 6 SMRT cells (Table 1). A circular chromosome was unambiguously assembled by trimming and joining the overlapping 3′ and 5′ ends from three large contigs of 3,866,718 bp, 715,826 bp and 541,428 bp, respectively. Contig joins were confirmed by PCR. Previously, we showed that a 14 scaffold draft 454 genome assembly of *E. coli* EC958 contained two additional replicons: a large antibiotic resistance plasmid (pEC958) and a small high-copy cryptic plasmid (pEC958B) [7]. In the PacBio assembly we found that pEC958 was represented as single circular contig of 135,602 bp that was consistent with the pEC958 scaffold in the original draft assembly (scaffold HG328349). In contrast, pEC958B was too small to be assembled using the HGAP parameters employed for rest of the chromosome, but it could be assembled from PacBio reads using a read-mapping approach.

**Figure 1:**
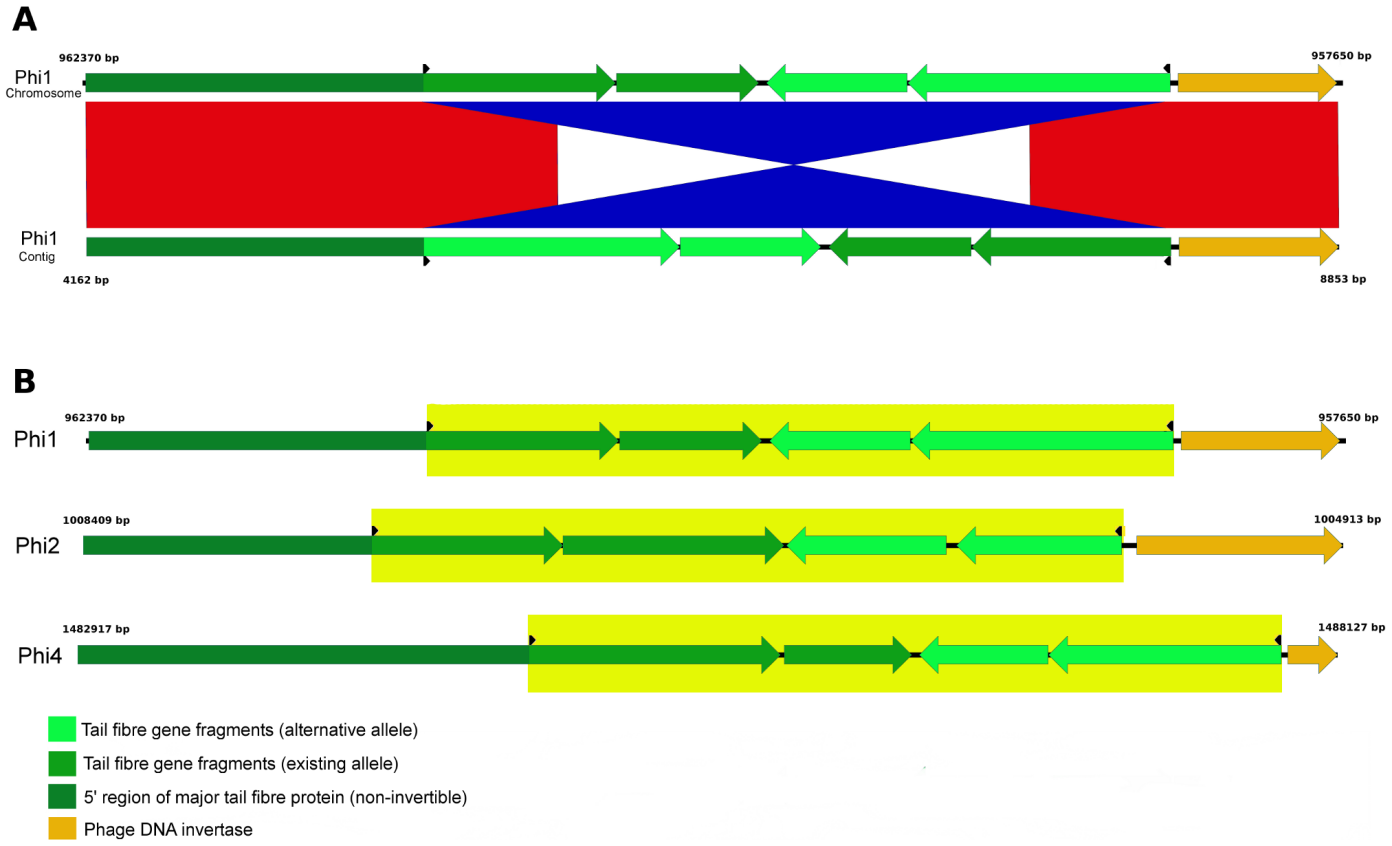
Prophage tail fibre allele switching in EC958. **A.** Alignment of the Phi1 alternative contig that contains the inversion of the tail fibre region to the genome of EC958. Phage tail fibre genes are coloured from dark green to light green. Phage DNA invertase genes are coloured orange. 26 bp crossover sites are indicated by black arrows. Red shading indicates nucleotide identity in the same orientation. Blue shading indicates nucleotide identity in the opposite orientation, highlighting the inversion in the phage tail fibre region. **B.** Genetic loci map of the tail fibre gene region of EC958 phages (Phi1, Phi2 and Phi4) and the location of recombination sites for DNA invertase. The major tail fibre gene is formed by a fusion of the stable 5′ region (dark green), encoding a series of Phage_fibre_2 tandem repeats (Pfam03406), with the invertible 3′ region (green) that encodes a Phage Tail Collar domain (Pfam07484). Downstream and presumably co-transcribed with the major tail fibre gene is a minor tail fibre gene (green). The alternate alleles form a mirror image of this arrangement, immediately downstream of the functional phage tail genes (lime green), enabling a new major tail fibre gene (and cognate minor tail fibre gene) to be formed by inversion of a 2–3 kb DNA segment. DNA invertase genes are coloured orange. The Phi4 prophage encodes a truncated DNA invertase (EC958_1582) that lacks the characteristic helix-turn-helix resolvase domain (PF02796). Invertible regions are highlighted in yellow. Figure prepared using Easyfig [27].

The contig order and orientation in the original draft 454 assembly was contiguous with the complete PacBio assembly determined in this study. We also found a high degree of consensus concordance between the two technologies with only fifteen single nucleotide indels and a single substitution between the two assemblies, most of which could be accounted for by homopolymeric tract errors in the 454 assembly according to comparisons with independent *E. coli* genomes and manual read inspection (Table 2). We also noted two discrepant regions that exhibited a cluster of substitutions and indels in the GI-*leuX* genomic island and in the tail fibre region of prophage Phi1 that initially appeared to be PacBio assembly errors. Further investigation revealed that the GI-*leuX* discrepancies were within a 3727 bp repeat region also found within GI-*selC*, thus the differences were due to a collapsed repeat in the 454 assembly (Table 2). In contrast, the Phi1 prophage discrepancy corresponded to a 2773 bp segment in the tail fibre region that was also present in an inverted orientation within a separate 12.2 kb contig (Fig. 1A). This spurious contig resulted from the assembly of PacBio reads (approximately 50% of all reads in this region) that contained the 2.8 kb segment in an alternative orientation, suggesting that high-frequency allele switching had occurred during propagation of *E. coli* EC958 prior to DNA extraction. Prophage tail fibre allele switching mediated by a site-specific DNA invertase has long been recognised as a phenomenon for altering host specificity of phage by alternating in-frame C-terminal phage tail fibre protein fragments (for review see Sandmeier, 1994 [46]). Interestingly, we also identified PacBio contigs corresponding to alternative alleles of prophage tail fibre regions from prophage Phi2 and Phi4 that were separately assembled into 8.7 kb and 12.7 kb contigs, respectively, due to 2–3 kb inversions (Fig. 1B). SMRTbell adapter sequences were found to be present in only 620 of 217,502 subreads (0.29%). This low level of adapter contamination combined with the absence of any hairpin artefacts at contig break points make it highly unlikely that aberrant reads are responsible for the three small phage-associated contigs, and suggest these contigs represent real biological variation of tail fibre genes in the chromosome of EC958. All three invertible segments exhibited the 5′ and 3′ 26 bp crossover sites characteristic of DNA invertase mediated phage tail switching mechanisms [46] (Table 3).

**Table 2:** Comparison of complete PacBio EC958 genome with draft 454 EC958 genome.

	Variant			454 contig information^2^				
Position^1^	PacBio	454	SE15	Name	Position	Length	Genomic context	Comment^3^
786693	.	A	.	00007	131223	131239	Intergenic (EC958_0852 and EC958_0854)	Homopolymeric tract
955837–957967	N/A	N/A	N/A	00011	62258	64195	Phi1 phage tail region	2.8 Kb invertable region in Phi1 phage tail region (2 substitutions/20 indels)
985718	T	.	T	00014	19202	34445	Intergenic (EC958_1049 and EC958_1050)	Homopolymeric tract
1493217	.	T	T	00033	1143	74077	Tryptophan biosynthesis protein TrpCF (EC958_4924)	454 variant consistent with SE15 genome
2027391	A	.	A	00040	28390	75386	Chemotaxis protein CheA (EC958_2110)	Homopolymeric tract
2598139	A	.	A	00085	32434	57547	Intergenic (EC958_2623 and EC958_4988)	Homopolymeric tract
3098765	A	.	A	00073	3745	4934	Hypothetical protein (EC958_5038)	Homopolymeric tract
3377055	.	C	.	00057	28348	88502	Hypothetical protein (EC958_5205)	Homopolymeric tract
4149057	A	.	A	00104	7234	41331	Type II restriction enzyme (EC958_4083)	Homopolymeric tract
4308872	T	.	T	00148	3658	51318	Intergenic (EC958_4231 and EC958_4232)	Homopolymeric tract
4380208	T	.	T	00146	7992	42111	Hypothetical protein (EC958_4927)	Homopolymeric tract
4756303	A	.	A	00117	4118	15191	Intergenic (EC958_4610 and EC958_4611)	Homopolymeric tract
4762264	T	.	T	00117	10078	15191	Hypothetical protein (EC958_5122)	Homopolymeric tract
4762871	A	.	.	00117	10684	15191	Hypothetical protein (EC958_5123)	Homopolymeric tract; 454 variant consistent with SE15 genome
4776778	A	.	A	00119	7363	155673	Transcriptional activator CadC (EC958_4623)	Homopolymeric tract
4938457	G	T	G	00158	1028	1512	Transposase DDE domain protein (EC958_5125)	1.5 Kb repeat region duplicated in pEC958
4963207–4965562	N/A	N/A	N/A	00105	1144	3727	Repeat region in GI-LeuX	3.7 Kb repeat region duplicated in GI-SelC (11 substitutions/4 indels)

1 Nucleotide position (or range) in complete PacBio EC958 genome.

2 Name, position of variant and length of 454 contig from draft 454 assembly.

3 “Homopolymeric tract” indicates that variant falls within tract of 5 or more nucleotides of same type.

**Table 3:** Sites of DNA inversion within EC958 prophage genomes as determined by PacBio assembly of alternate alleles.

Crossover site	Sequence^1^	Location^2^	Comments^3^
Phi1_5prime	[gccg**TT**A**TCGAA**T**ACCTC**∧**GGTTTACGAGAA** - 478 bp]	c961070..961095	Part of 508 bp imperfect inverted repeat (77% nt identity); 2773 bp invertible segment
Phi1_3prime	[gcca**TT**A**TTTAAAACCTC**∧**GGTTTACGAGAA** - 478 bp]	958322..958347	-
Phi2_5prime	[ **T**C**CTCAA**TT**ACCTT**∧**GGTTTAGGAGAA** - 197 bp]	c1007582..1007607	Part of 227 bp imperfect inverted repeat (96% nt identity); 2067 bp invertible segment
Phi2_3prime	[GAGAG**ATAAACGTT**∧**GGTTT**G**GGGGAA** - 197 bp]	1005540..1005565	-
Phi4_5prime	[ccgccg**TT**A**TCGAA**T**ACCTC**∧**GGTTTACAGGAA** ]	1484784..1484809	Part of 36 bp imperfect inverted repeat (3 mismatches); 3106 bp invertible segment
Phi4_3prime	[ccgcca**TT**A**TCTAAAACCTC**∧**GGTTTACGAGAA** ]	c1487865..1487890	-
**Consensus**	**TTCCC.TAAACGTT∧CGTTTA.AAGAA**	n/a	Based on consensus of crossover sites from.
	**TT.A C C G T.GG**		Mu, P1, e14, p15B and *S. boydii* DNA inversion systems, as previously determined by Sandmeier et al. 1994 [42]

1 Predicted binding site for DNA invertase shown in capital letters; site of strand exchange is indicated by underlined central dinucleotide with ∧ indicating downstream staggered cut; nucleotides in bold are consistent with the previously determined consensus DNA invertase crossover site [42]; square brackets indicate boundaries of larger imperfect inverted repeats that encode the crossover sites.

2 Coordinates refer to start and end of 26 bp crossover site in EC958 complete genome; 5prime/3prime orientation is relative to the complete prophage tail fibre gene and prophage genome; c = complement.

3 Phi1 and Phi4 5prime and 3prime 26 bp crossover sites differ by only 2 and 1 mismatches, respectively.

### *E. coli* EC958 general genome features

The genome of *E. coli* EC958 consists of a single circular chromosome of 5,109,767 bp with an average GC content of 50.7%. The chromosome encodes 4982 putative protein-coding genes, including 358 that were not previously annotated on the draft chromosome due their presence in repetitive regions that were not assembled as scaffolds. Seven rRNA loci, consisting of 16S, 23S and 5S rRNA genes, and 89 tRNA genes, representing all 20 amino acids, were identified on the chromosome. As described elsewhere [7], the virulence-associated gene complement of EC958 includes adhesins (e.g. *fimA-H*, *afa* and *curli*), autotransporters (e.g. *agn43*, *upaG*, *upaH*, *sat* and *picU*), iron receptors (e.g. *fepA*, *iutA*, *iha*, *chuA*, *hma* and *fyuA*) and a number of other virulence associated genes (e.g. *kpsM, usp, ompT, malX*). Four genes that were not annotated in the draft genome may be virulence related: *sitB* (EC958_5193), which encodes a component of an iron transport system that is up-regulated during *Shigella* intracellular growth [47]; and three hypothetical genes (EC958_4894, EC958_4977, EC958_4981) orthologous to genes previously identified as uropathogenic *E. coli* specific [48]. The EC958 large plasmid, pEC958, is predicted to contain 151 protein-coding genes, including a 22 kb locus encoding conjugal transfer (*tra*) genes and antibiotic resistance genes including *bla*_CTX-M-15_ [7].

### Whole genome comparison of *E. coli* EC958, NA114 and SE15

**Figure 2:**
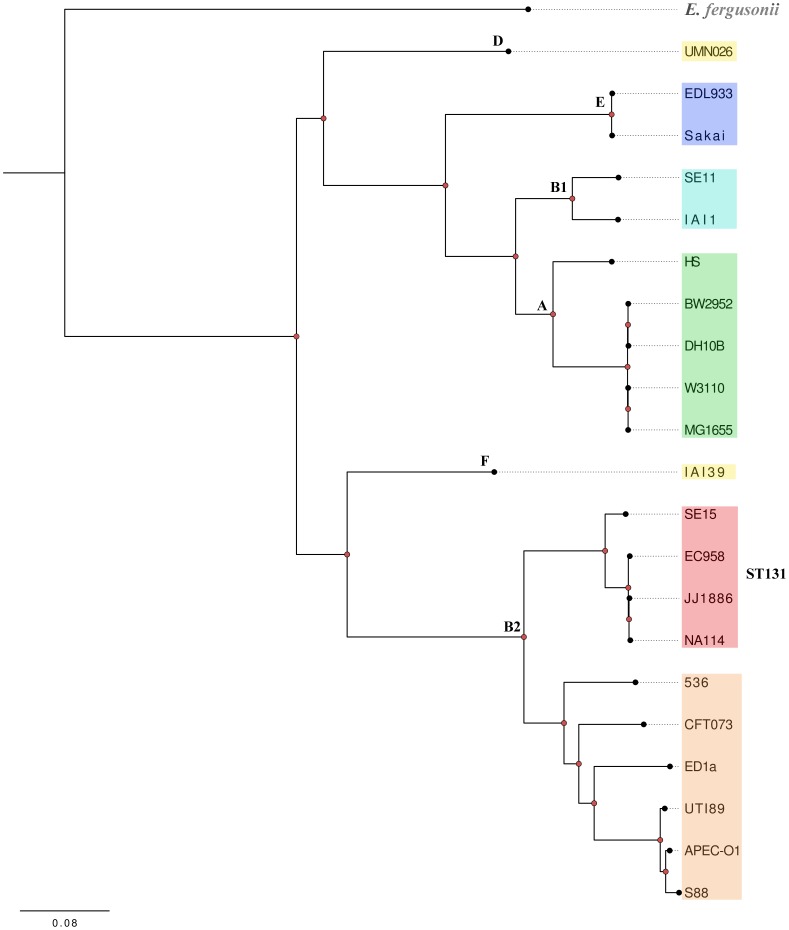
Maximum likelihood phylogenetic comparison of 4 ST131 and 17 representative *E. coli* isolates. The tree is rooted using the out-group species *E. fergusonii* ATCC35469. The phylogenetic relationships were inferred with the use of 261,214 SNPs identified between the genomes of the 22 *Escherichia* strains and 1000 bootstrap replicates. The major *E. coli* phylogroups are coloured as follows; phylogroup B2-ST131: SE15, NA114, JJ1886, EC958 (red); other phylogroup B2: APEC-01, S88, 536, UTI89, CFT073, ED1A (orange); phylogroup D: UMN026 (yellow); phylogroup F: IAI39 (yellow); phylogroup A: BW2952, MG1655, W3110, HS (green); phylogroup B1: SE11, IAI1 (aquamarine); phylogroup E: O157 EDL933, O157 Sakai (blue). Red nodes have 100% bootstrap support from 1000 replicates.

**Figure 3:**
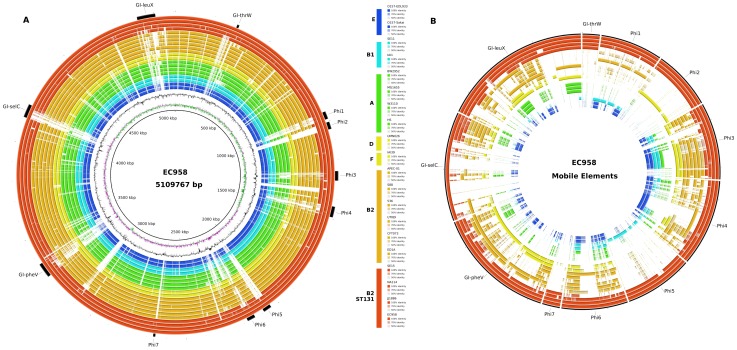
Distribution of EC958 mobile genetic elements in *E. coli*. **A.** Visualisation of the EC958 genome compared with three *E. coli* ST131 genomes and 16 other *E. coli* genomes using BLASTn. EC958 prophage (Phi1 – Phi7) and genomic islands (GI-*thrW*, GI-*pheV*, GI-*selC*, GI-*leuX*) are represented by black boxes in the outermost circle. The innermost circles represent the GC content (black) and GC skew (green/purple) of EC958. The remaining circles display BLASTn searches against the genome of EC958. **B.** A BRIG visualisation of the EC958 mobile elements compared with the 19 *E. coli* genomes. BLASTn searches of the 19 genomes against the EC958 prophage and genomic islands show that the EC958 GIs and prophage are well conserved in the ST131 clade C genomes but largely absent from the genomes of SE15 and the other 16 *E. coli* genomes, which are arranged inner to outer as follows: Group E strains O157 EDL933, O157 Sakai (blue); group B1 strains SE11, IAI1 (aquamarine); group A strains BW2952, MG1655, W3110, HS (green); group D strains UMN026, IAI39 (yellow); group B2 strains APEC-01, S88, 536, UTI89, CFT073, ED1A (orange); group B2 ST131 strains SE15, NA114, JJ1886, EC958 (red). Figure prepared using BRIG [28].

Phylogenetic analyses indicated that *E. coli* strains EC958, NA114 and JJ1886 cluster together in a clade discrete from *E. coli* SE15 within an ST131 specific lineage within the B2 phylogroup (Fig. 2). Whole-genome BLASTn comparisons showed that the major structural differences between the genomes of SE15 and the three *fimH30* ST131strains relate to the seven prophage loci (Phi1-Phi7) and four genomic islands (GI-*thrW*, GI-*pheV*, GI-*selC*, and GI-*leuX*) that were previously defined in the draft genome of *E. coli* EC958 [7] (Fig. 3A). The complete PacBio genome confirmed the position and size of these elements and was able to fill numerous gaps caused by insertion elements or other repetitive elements. These prophage and GI regions are absent in whole or in part from *E. coli* SE15, and from most of the 16 other *E. coli* representative strains surveyed (Fig. 3B). Additionally, GI-*selC* is largely absent from all ST131 strains except EC958, whereas GI-*thrW* and Phi7 are well conserved in all four ST131 strains (Fig. 3B). Genomic surveys with a greater number of ST131 strains from diverse origins will be necessary to determine the prevalence of prophage, genomic islands and other mobile genetic elements.

### Large discrepancies between ST131 genomes are likely due to misassembly of *E. coli* NA114

**Figure 4:**
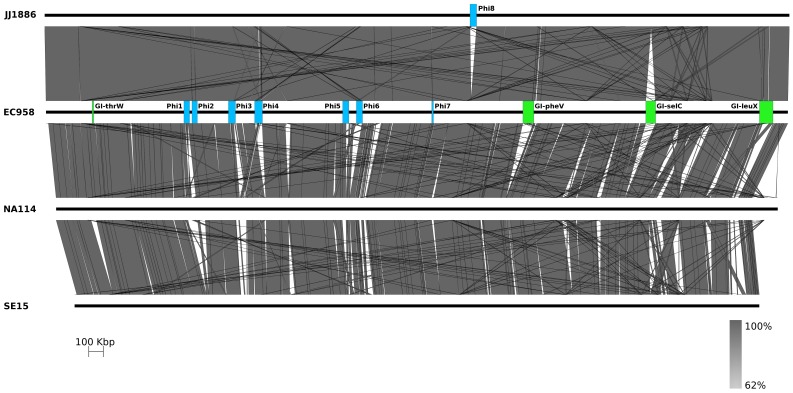
Nucleotide pairwise comparison of four *E. coli* ST131 chromosomes showing extensive variation in the structure and location of EC958 prophage elements (blue) and genomic islands (green). An additional prophage element present in JJ1886 has also been annotated here as Phi8 for clarity. ST131 genomes are arranged from top to bottom as follows: JJ1886, EC958, NA114, SE15. Grey shading indicates nucleotide identity between sequences according to BLASTn (62%–100%). Figure prepared using Easyfig [27].

**Figure 5:**
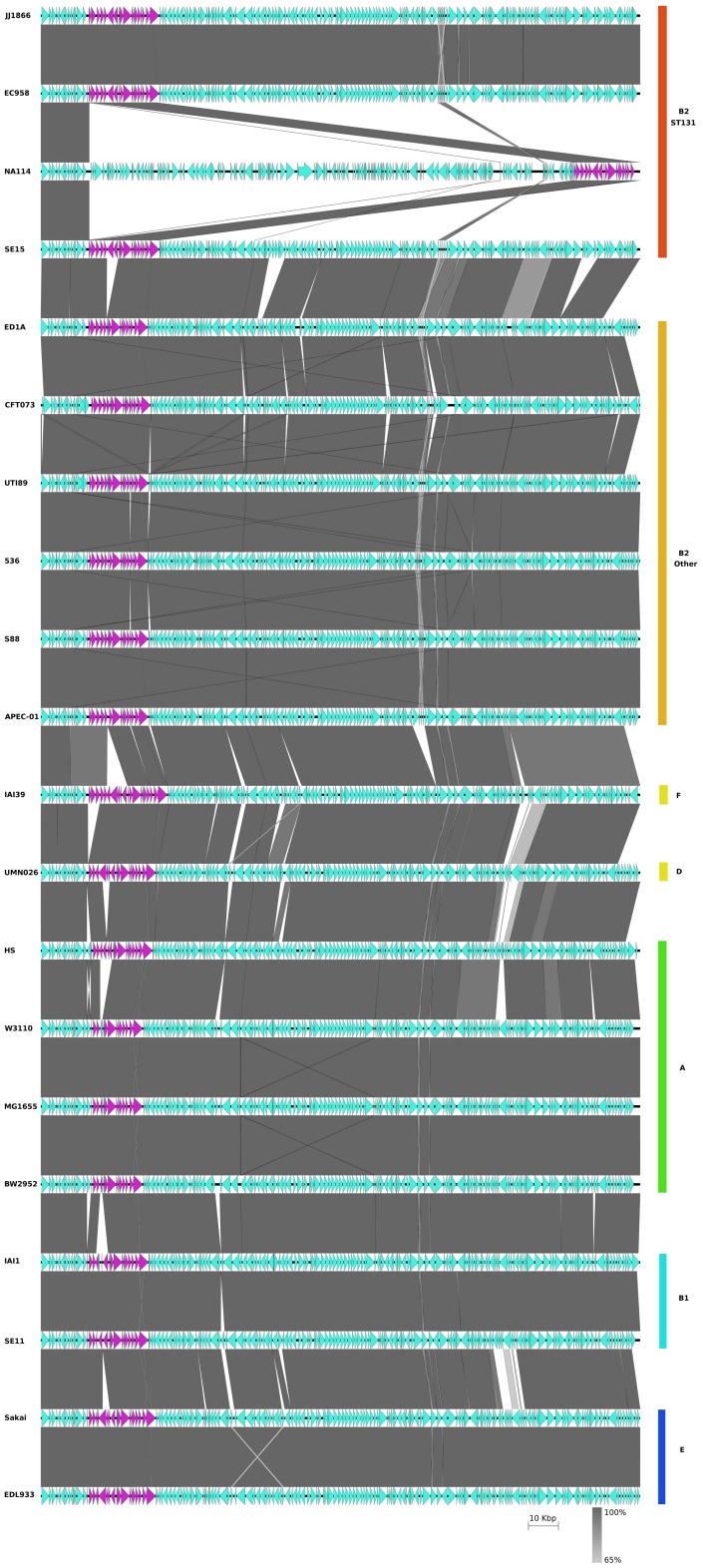
Nucleotide pairwise comparison of a 200 kb region (*thrA* to *degP*) from the genomes of the four ST131 and 16 other representative *E. coli* strains. Grey shading indicates nucleotide identity between sequences according to BLASTn (62%–100%). Coding regions immediately upstream of *dnaJ* are highlighted in purple. This region is well conserved in 19 of 20 *E. coli* genomes examined. However, a large insertion in the genome of NA114 located immediately upstream of *dnaJ* is clearly evident (white). *E. coli* genomes are arranged from top to bottom as follows: group B2 ST131 strains JJ1886, EC958, NA114, SE15 (red); group B2 strains ED1A, CFT073, UTI89, 536, S88, APEC-01 (orange); group F strain: IAI39 (yellow); group D strain UMN026 (yellow); group A strains HS, W3110, MG1655, BW2952 (green); group B1 strains IAI1, SE11 (aquamarine); group E strains O157 Sakai, O157 EDL933 (blue). Figure prepared using Easyfig [27].

At the core genome level EC958, NA114, JJ1886 and SE15 all display a high level of genome synteny, with major differences due to the number, content and location of integrated mobile elements giving rise to variation in chromosome length (Fig. 4). Whereas *E. coli* EC958 and *E. coli* JJ1886 chromosomes are 5.10 Mb and 5.12 Mb, respectively, *E. coli* NA114 is almost 200 kb smaller at 4.9 Mb, and *E. coli* SE15 has a 4.7 Mb chromosome. In addition to all seven defined EC958 prophages, the JJ1886 chromosome possess an additional prophage (Phi8) not present in the genomes of the other ST131 strains, but otherwise exhibits a high degree of synteny with the EC958 chromosome (Fig. 4). In contrast, the chromosome of *E. coli* NA114 shows multiple gaps relative to EC958, exhibits significant variation in both the number and content of prophages, and appears to lack the three largest defined EC958 genomic islands (GI-*pheV*, GI-*selC* and GI-*leuX*) (Fig. 4). Instead, *E. coli* NA114 has a ∼160 kb region immediately upstream of *dnaJ* that consists of an assortment of GI and prophage sequence fragments that are found in several different locations in the EC958 and JJ1886 genomes. The *dnaJ* locus is not a known genomic island integration site and is well conserved in *E. coli* genomes from all phylogroups (Fig. 5). Together, these observations suggested to us that the *E. coli* NA114 genome has been misassembled.

**Figure 6:**
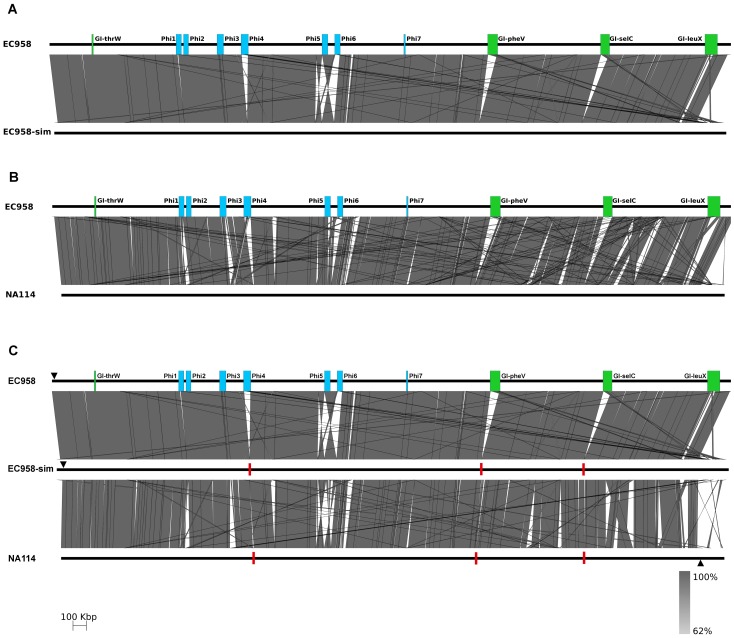
Nucleotide pairwise comparison between EC958, a simulated EC958 Illumina assembly and NA114. **A.** Nucleotide pairwise comparison of the EC958 chromosome (top) and a simulated EC958 chromosome assembly (EC958-sim, bottom). Linear alignments revealed extensive variations in the location and structure of mobile elements in EC958-sim when compared to EC958. Grey shading indicates nucleotide identity between sequences according to BLASTn (62%–100%). Prophage regions are annotated as blue boxes and genomic islands as green boxes. **B.** Nucleotide pairwise comparison of EC958 chromosome (top) and NA114 chromosome (bottom). **C.** Nucleotide pairwise comparison of EC958 (top), EC958-sim (centre) and NA114 (bottom) chromosomes. EC958 prophage and genomic islands misassembled in EC958-sim are similarly misassembled in the genome of NA114 (red boxes). Red boxes indicate positions in EC958-sim and NA114 where mobile genetic elements are present in EC958. The *dnaJ* gene is shown as a black triangle on each chromosome. Figure prepared using Easyfig [27].

To determine how a misassembly might have occurred, we replicated the NA114 assembly strategy and reassembled the genome of *E. coli* EC958 using simulated, error free, Illumina reads ordered against the *E. coli* SE15 chromosome (EC958-sim). We found that GI-*pheV*, GI-*selC*, GI-*leuX* and several of the prophage loci were placed incorrectly in EC958-sim relative to the complete *E. coli* EC958 genome (Fig. 6A). As expected, contigs associated with the EC958 genomic islands and prophages, which represent novel regions in the genome of EC958 compared to SE15, could not be correctly placed/ordered by alignment to SE15. Instead, these contigs have been randomly placed at the “end” of the chromosome in what might be mistaken for a large genomic island. Interestingly, the pattern of variation observed in the structure and location of EC958-sim mobile elements is similar to that observed in linear alignments of EC958 and NA114 (Fig. 6B and Fig. 6C). Of the 77 gaps observed when EC958-sim contigs (>200 bp) were aligned with the complete *E. coli* EC958 chromosome, the majority corresponded with deletions or rearrangements at corresponding positions in the *E. coli* NA114 chromosome (Fig. 6C and Dataset S1).

## Discussion

Here we report the complete genome sequence of the *E. coli* ST131 strain EC958. Sequencing the genome of *E. coli* EC958 with six SMRT cells of data followed by *de novo* assembly using the HGAP method and minimal post-processing produced a high quality finished genome comparable in terms of contiguity and error rate with a 454 GS-FLX mate-pair derived assembly. Since the sequence data for this genome was generated, the PacBio SMRT platform has transitioned from the RS I to the RS II instrument and improved chemistry, with average read lengths increasing to ∼8 kb. Consequently, we expect that sequencing strategies utilising fewer than six SMRT cells on the PacBio RS II platform should be capable of producing fully assembled bacterial genomes with minimal intervention.

The sensitivity of PacBio for detecting dynamic prophage rearrangements is due to the length of PacBio reads, which allows them to span inverted regions and thus force the assembler to generate two alternative versions of regions that have undergone inversion in a subset of the bacterial population. In contrast, such mixed inversions are more difficult to detect in shorter read assemblies, which would normally require separate mapping and detection of discordant read-pairs to identify. Although there have been no other reports of phage tail inversion in PacBio assemblies to date, others have noted that a ∼7.5 kb “spurious contig” was produced in the assembly of the *E. coli* K-12 MG1655 genome [23]. PacBio thus offers a novel solution for studying the mechanism of phage tail fibre switching, and more generally, for the function of DNA invertase and other site-specific recombinases. For example, the DNA invertase gene has been severely truncated in the Phi4 prophage, suggesting that the inversion observed in this study must have been mediated by another enzyme *in trans*, as has been previously reported [49]–[51]. Notably, the Phi1 and Phi4 prophages encode near-identical 26 bp crossover sites at either end of their respective invertible segments (Table 3), suggesting that the Phi1 DNA invertase may be capable of mediating inversion at heterologous sites within the Phi4 prophage.

On a practical level, users should ensure that alternative allele contigs in PacBio assemblies are not integrated into the assembly of the main chromosome, which would lead to artefactual duplications in phage regions. Instead, we have annotated the EC958 chromosome to highlight the DNA invertase binding sites and invertible regions with misc_feature keys according to INSDC guidelines. We have also simplified the annotation of these regions to help avoid propagating genome-rot in *E. coli* genomes; for example, alternate phage tail gene 3′ fragments that contain the Phage Tail Collar domain but lack the Phage Tail Repeat domains are often auto-annotated as “Phage tail repeat domain proteins” due to their similarity to their full-length homologs. For *E. coli* assemblies, it is relatively straight-forward to determine which contigs are alternate versions of inverted loci as opposed to truly independent contigs, by first aligning all contigs to each other during post-assembly using tools such as ACT [25] or Contiguity (http://mjsull.github.io/Contiguity/). However, care must be taken to ensure that “recombination” is not due to adapter sequences. Due to the high error rates associated with raw PacBio reads, occasionally adapters on the ends of the SMRTbell construct are not correctly identified and removed [52]. Failure to remove adapter sequences can result in chimeric subreads which consist of the insert sequence in the forward orientation followed by the adapter sequence and the insert sequence in the reverse orientation. Adapter sequences occur randomly within the reads and are removed during read correction but aberrant reads can be produced. Retaining these reads can result in false hairpins in assemblies and the generation of small spurious contigs. Users should also be aware that small plasmids are not necessarily assembled from PacBio reads using seed read length cut-offs in excess of the total plasmid size, as illustrated in this study with the 4.1 kb pEC958B plasmid. In this case we assembled pEC958B by utilising prior knowledge of the plasmid from the original 454 assembly, however, *de novo* assembly of the entire genome would be possible by iteratively reducing the seed read length cut-off within HGAP (data not shown).

We previously generated a high-quality draft sequence of *E. coli* EC958 [7], however, using only PacBio reads we were able to assemble a high-quality complete genome sequence. A comparison of the complete PacBio and draft 454 assemblies revealed a small number of discrepancies, the majority of which were due to homopolymeric tracts in the 454 assembly or collapsed repeats that were resolved in favour of the PacBio consensus after closer inspection. Although contig order and orientation in the original draft assembly was contiguous with the PacBio assembly, only the latter was able to resolve repetitive regions of the genome such as rRNA operons, extended tracts of tRNAs, prophage loci and insertion sequences (IS) within the GI-*pheV*, GI-*selC* and GI-*leuX* genomic islands. The long, multi-kilobase reads produced in SMRT sequencing can be unambiguously anchored with unique sequences flanking these repeats, allowing for their accurate and uninterrupted assembly. Given the rapid improvements in PacBio technology, and the HGAP assembly software [23], this technology may become the platform of choice for generating high-quality reference sequences for bacterial genomes.

Comparisons of the complete *E. coli* EC958 genome against other published ST131 genomes revealed the extensive nucleotide identity that exists between the core genomes of *E. coli* ST131 clade C strains EC958, NA114 and JJ1886. Although *E. coli* NA114 possesses many of the genes associated with genomic islands and prophages of EC958 and JJ1886, it lacks insertions at recognised *E. coli* integration hotspots, including the *pheV* tRNA gene [28]. Furthermore, it contains a highly atypical insertion of ∼160 kb within a location that is consistent with the artefactual concatenation of contigs, “junked” at the end of the assembly, that could not be ordered against the SE15 reference genome. Our recent comparative genomic analysis has shown that, with the exception of GI-*selC* and Phi6, the genomic islands and prophages previously defined in EC958 are prevalent in nearly all other ST131 clade C strains [21]. Based on our whole genome comparisons of EC958, NA114, JJ1886 and SE15, and our simulated draft Illumina assembly (EC958-sim), we suggest that much of the variation in mobile elements observed between NA114, EC958 and JJ1886 is not biologically relevant but rather the result of systematic errors introduced during the assembly of the *E. coli* NA114 genome.

Genome misassemblies are not only confined to draft genomes and have previously been identified in finished genomes [15]. Furthermore, in recent years a number of draft genomes have been erroneously deposited into the complete genome division of GenBank/EMBL/DDBJ, with reversal of sequence deposition very difficult due to the structure of these databases. Due to the clinical importance of uropathogenic *E. coli* we believe it is important to bring the misassembly of the *E. coli* NA114 genome to the attention of the community, particularly as it has been used recently in genome comparisons as if it was complete [22], and was used as the reference genome in a larger study of 100 *E. coli* ST131 isolates [6]. It should be more broadly recognised that it is not possible to generate an accurate representation of a complete *E. coli* genome by *de novo* assembly of Illumina, 454 or Ion Torrent reads alone. Ideally, a combination of paired-end and mate-pair libraries of varying insert length, often combined with PCR/Sanger sequencing, is necessary to correctly place contigs generated by SGS technologies and accurately close the gaps between them. In contrast, we show here that PacBio is able to act as a stand-alone platform for the generation of high-quality complete bacterial genome sequences. The availability of a complete, annotated genome of *E. coli* EC958 will provide an important resource for future comparative studies and reference guided assemblies of *E. coli* ST131 clade C/*fimH30* genomes.

## Supporting Information

Dataset S1 **Genome sequences of EC958, EC958-sim and NA114 and BLASTn comparison files required to create an ACT image as seen in** **figure 6C** **.**
(ZIP)
